# Synthesis of Acrylic–Urethane Hybrid Polymer Dispersions and Investigations on Their Properties as Binders in Leather Finishing

**DOI:** 10.3390/polym17030308

**Published:** 2025-01-24

**Authors:** Selime Keskin, Catalina N. Cheaburu-Yilmaz, Aylin Altinisik Tagac, Raluca Nicoleta Darie-Nita, Onur Yilmaz

**Affiliations:** 1 Leather Engineering Department, Faculty of Engineering, Ege University, Bornova, Izmir 35100, Türkiye; selime.keskin@herkimgroup.com; 2 FARBEN Deri ve Kimyevi Maddeler San. Tic. A.Ş., İstanbul Deri OSB. Pres Sokak No:3, Tuzla, Istanbul 34956, Türkiye; 3 Chemistry Department, Faculty of Science, Dokuz Eylul University, Tınaztepe Campus, Buca, Izmir 35390, Türkiye; catalinanatalia.yilmaz@deu.edu.tr (C.N.C.-Y.); aylin.altinisik@deu.edu.tr (A.A.T.); 4 Center for Fabrication and Application of Electronic Materials, Dokuz Eylul University, Buca, Izmir 35390, Türkiye; 5 Physical Chemistry of Polymers Department, “Petru Poni” Institute of Macromolecular Chemistry, 41A Gr. Ghica Voda Alley, 700487 Iasi, Romania

**Keywords:** polyurethane, acrylic, hybrid polymer, seeded emulsion, miniemulsion, leather finishing

## Abstract

This study investigates the synthesis and application of acrylic–urethane hybrid polymer dispersions as advanced binders for leather finishing. Two polymerization techniques—seeded emulsion and miniemulsion—were used to produce hybrid polymer dispersions by varying the ratios of polyurethane (PU) and acrylic (AC). The synthesized dispersions, i.e., the hybrid polyurethanes, showed stable, uniform particle sizes, inferring good compatibility and interaction between the PU and AC phases, as confirmed by particle sizes, FTIR, and DSC analyses. The performance of the coating on leather surfaces was assessed by using standard physical tests, including rubbing fastness, flexing endurance, water spot resistance, and grain strength. The results showed that the hybrid polymers outperformed their individual PU and AC counterparts, particularly in terms of abrasion resistance and mechanical integrity. Of the two polymerization techniques, the seeded emulsion hybrids exhibited superior coating properties, providing greater resistance to cracking and abrasion under stress, improved grain strength, and better color retention during rubbing tests. These findings highlight the potential of acrylic–urethane hybrids, particularly those prepared via seeded emulsion polymerization, to address the limitations of traditional binders in high-performance leather applications.

## 1. Introduction

Leather finishing is a sequential process performed in the final phase of leather manufacturing, which plays a crucial role in defining the ultimate properties of leather goods. This stage determines characteristics such as appearance, coverage of surface defects, gloss, feel, color uniformity, trend effects, patterns, and resistance to mechanical stress and weathering. The primary component of a leather-finishing system is the binder, as it forms a coating on the leather surface and binds the colorants and other chemicals to the substrate. Polyurethane (PU) and acrylic dispersions are the main types of binders used in leather finishing [1].

Polyacrylates exhibit strong performance, including high block resistance, a balance of hardness and softness, good adhesiveness, excellent film-forming properties, UV resistance, and cost efficiency [2]. However, their limitations include low chemical and water resistance, as well as reduced weathering stability and mechanical strength. In contrast, waterborne polyurethanes (WPUs) excel in adhesion, elasticity, flexibility, and abrasion resistance, but are hampered by high costs, low pH stability, and limited durability for outdoor applications [3,4,5,6]. In leather finishing, blends of acrylic and polyurethane binders are often used to reduce costs while leveraging the advantages of both. However, when high physical and chemical performances are required, such blends may not fully meet the necessary specifications. Performance reductions are often more pronounced than expected, potentially due to incompatibilities between the polymers, leading to phase separation during film formation [7].

A promising alternative involves combining the properties of both types of polymers in a single material, referred to as a “hybrid”. Hybrid materials are expected to provide superior performance compared to blends of acrylic latexes and emulsified polyurethanes [8,9]. In general, two main methods are used to produce PU–acrylic hybrid particles [10,11].

The first method involves dissolving an NCO-terminated PU prepolymer in an acrylic monomer, followed by dispersion using shear forces and an external emulsifier to create a miniemulsion. The acrylic phase is polymerized by a typical miniemulsion process, during which PU chains are extended. For example, Wang et al. (2005) investigated the synthesis and properties of acrylic–polyurethane hybrid latexes prepared by miniemulsion polymerization, comparing these hybrids to simple blends of the same components. In their method, PU was synthesized using isophorone diisocyanate and polypropylene glycol, with butane diol as a chain extender. This PU was then miniemulsified with methyl methacrylate (MMA) and butyl acrylate (BA) and stabilized using sodium dodecyl sulfate (SDS). The resulting hybrids exhibited stability and improved mechanical properties, such as higher yield stress and elongation, compared to the blends. In addition, TEM and AFM analyses revealed that the hybrids had a more homogeneous structure with reduced phase separation, in contrast to the blends, which displayed distinct phase boundaries with higher PU contents [8]. Similarly, Udagama et al. (2011) developed acrylic–PU hybrid latexes using miniemulsion polymerization for pressure-sensitive adhesive (PSA) applications. Their approach began with the reaction of a low-molecular-weight isocyanate-terminated PU prepolymer with 2-hydroxyethyl methacrylate (HEMA), introducing methacrylic groups to allow subsequent copolymerization with acrylic monomers. Bisphenol A (BPA) was incorporated as a chain extender, and the miniemulsions were stabilized with surfactants and co-stabilizers prior to sonication. Polymerization was then carried out using n-butyl acrylate, methyl methacrylate, and acrylic acid, with a redox initiator system used to minimize side reactions. This method produced latexes with high solid contents, stable dispersions, and uniform particle sizes of about 100 nm, resulting in excellent adhesion and cohesive properties [12]. Other studies have highlighted the advantages of miniemulsion polymerization for producing uniform, stable hybrids with good interphase compatibility, although this approach requires high-energy emulsification, which can be challenging for industrial applications [13,14].

The second method uses seeded emulsion polymerization, where a PU dispersion is prepared—sometimes with an acrylic monomer—and then used as a seed for acrylic polymerization. This approach is more industrially feasible because it avoids high-energy emulsification steps, although control of the dispersion process remains a challenge. For instance, Athawale and Kulkarni (2009) investigated the preparation and properties of urethane–acrylate composites using semibatch emulsion polymerization. They synthesized hybrid latexes by combining acrylic monomers (styrene, butyl acrylate, and acrylic acid) with PU dispersions and compared them to physical mixtures of acrylic emulsions and PU dispersions. Varying the acrylic-to-PU ratio revealed enhanced performance and improved microphase structures in the hybrids. Characterization by FTIR and TGA demonstrated superior compatibility and significantly improved chemical and mechanical properties in hybrids compared to blends, with the optimal 50:50 (w/w) ratio yielding the best coating performance [15]. In another study, Puyadena et al. (2022) developed phosphorus-modified PU–acrylic hybrid dispersions for flame-retardant coatings. The PU prepolymer was synthesized using isophorone diisocyanate, polycaprolactone diol, polyethyl-phosphate glycol ester, and dimethylpropionic acid, with methyl methacrylate as a solvent. The phosphate ester of 2-hydroxyethyl methacrylate was incorporated to end-cap the chains with acrylic moieties. After neutralization and emulsification in water, the acrylic phase was polymerized by free radical polymerization. The study highlighted the challenges in achieving homogeneous and stable dispersions, requiring optimization of monomer types and ratios [16]. Numerous studies have reported the use of seeded polymerization for the preparation of urethane–acrylic polymer hybrid dispersions [17,18,19,20,21,22]. Achieving stable and homogeneous dispersions often requires the preparation of a prepolymer with an ionizable moiety, the use of a solvent to reduce viscosity for effective dispersion, and the addition of stabilizers or emulsifiers before or after acrylic-phase polymerization.

Few studies in the literature address the preparation and application of urethane–acrylic polymer hybrid dispersions for leather finishing. Krings et al. (2010) from Stahl Co. (Waalwijk, The Netherlands) highlighted the potential benefits of acrylic–urethane hybrid polymers in leather finishing, reporting superior cold flex endurance, rubbing fastness, and hydrolysis resistance compared to traditional PU or their blends with acrylics. However, they did not provide details about the preparation methods [7].

Chai and Zhang (2010) synthesized various PU and PU–polyacrylate (PUA) composite emulsions using seeded emulsion polymerization. Using glycidyl methacrylate to bond the core–shell layers, they produced core–shell and interpenetrating emulsions with enhanced film-forming features, adhesion, flexing properties, and improved rubbing resistance for leather finishing [17].

Ryu et al. (2016) developed waterborne PU–acrylic hybrid binders without emulsifiers and solvents, examining the effects of the glycidyl methacrylate/acrylonitrile ratio on particle size, viscosity, and stability. These binders have proven effective as adhesives between polyethylene sheets and synthetic leather [4,18].

The present study focuses on the synthesis and characterization of urethane–acrylic hybrid polymer dispersions using both seeded emulsion and miniemulsion polymerization techniques, with different ratios of PU and acrylic phases. The resulting hybrid emulsions were applied as single-binder systems in leather-finishing formulations, and their effectiveness in enhancing leather surface properties was evaluated through various standard physical tests. This research aims to determine the optimal polymer composition and polymerization method for improving the coating quality, durability, and overall performance of the finished leather.

## 2. Materials and Methods

### 2.1. Materials

For the synthesis of PU, dicyclohexylmethane-4,4‘-diisocyanate methylene-bis-(4-isocyanatocyclohexane) (H12MDI, Evonik (Essen, Germany), ≥99.5), polypropylene glycol-1000 (PPG1000, Bayer (Leverkusen, Germany), ≥99.5), polypropylene glycol-2000 (PPG2000, Bayer, ≥99.5), dimethylol propionic acid (DMPA, Bayer, ≥99.5), ethylene glycol dimethyl ether (DME, KH Chemicals B.V., Zwijndrecht, The Netherlands, ≥99.5), bismuth neodecanoate (BiN, Ege Kimya (Istanbul, Turkey), ≥99.5), triethyl amine (TEA, Ataman Kimya (Istanbul, Turkey), ≥99.7%), and hydrazine (Hyd, Ataman Kimya, ≥99.9) were used.

Monomers of methyl methacrylate (MMA, ≥98.5%), butyl acrylate (BA, ≥99.0%), and methacrylic acid (MAA, ≥99.0%) were sourced from Sigma-Aldrich (Schnelldorf, Germany) and utilized for the synthesis of the acrylic part (AC) of the hybrids. Sulfopon^®^ 101 UP (BASF, Ludwigshafen, Germany, 28–30%) and Emulsogen EPN 287 (Clariant (Muttenz, Swtizerland), 68–70%) were used to stabilize the emulsions. Sodium bicarbonate (NaHCO_3_, ≥99.0%), ammonium persulfate (APS, ≥98.0%), and hexadecane (HD, ≥99.0) were all acquired from Merck (Darmstadt, Germany) and applied as the buffering agent, initiator, and co-solvent, respectively.

All reagents were used as received, without further purification. Ultrapure water was used as a solvent for the reactions. The syntheses were carried out in a 250 mL four-neck round-bottom glass reactor, equipped with a condenser, dropping funnels, and a mechanical stirrer. Reaction conditions and formulation preparation were controlled using a temperature probe heater (IKA, Staufen, Germany) in combination with an oil bath.

The binders were applied to black-colored cattle crust leathers, suitable for footwear, and cut to A4 size. The leathers and other required finishing chemicals were supplied by FARBEN Co. (Istanbul, Turkey).

### 2.2. Polymerization Procedures

#### 2.2.1. Preparation of PU Prepolymer and Aqueous PU Dispersion

**Scheme 1:**
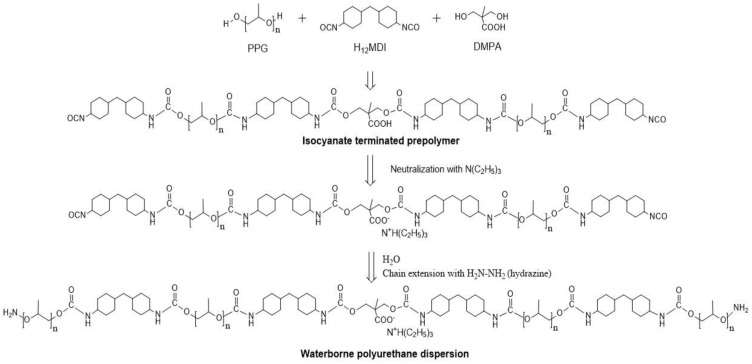
Synthesis of waterborne PU.

In the first step, the PU prepolymer was prepared using the reaction between H12MDI, PPG1000, PPG2000, and DMPA. The synthesis details are given in Table 1 and Scheme 1. First, the PPGs were loaded inside the reactor and heated up to 50 °C. DMPA and DME were then added and mixed until DMPA was completely dissolved; around 70 °C, the catalyst (BiN) and H_12_MDI were added in the reactor at 170 rpm and maintained for 4 h at a temperature of 85 °C. The NCO-terminated prepolymer was obtained at the end of the reaction.

In the second step, the reactor was cooled to 50 °C and TEA was added to neutralize the prepolymer. Hydrazine was then added as a chain extender followed by the addition of ice water to form the PU dispersion under vigorous stirring. Finally, a stable PU dispersion was obtained with a solid content of 25 ± 1 wt.% and a pH = 8.0 ± 0.5.

#### 2.2.2. Preparation of Acrylic Emulsion

A polyacrylic binder was also synthesized via the seeded emulsion polymerization technique for comparison purposes with hybrid polymers. The ratios of all ingredients used for the acrylic polymers were the same for all PU–AC hybrid preparations. For synthesis, the emulsifiers and NaHCO_3_ were dissolved in water at room temperature in the reactor and mixed for 15 min. An amount of 5 wt.% of the total monomer mixture composed of MMA, BA, and MAA was added in the reactor and mixed well to form the seed emulsion. The temperature was raised to 80 °C, and a portion of the APS solution was added to initiate the radical polymerization to form the seed polymer. After 15 min, the remaining monomer mixture and APS solution were continuously fed into the reactor for 2 h in separate funnels. After all transfers were completed, the system conditions were maintained for an additional 90 min to complete the reaction. Then, the reactor was cooled to room temperature and the pH was adjusted to 7.0 using triethanol amine. A stable blue- white polyacrylic latex was obtained with a solid content of 25 ± 1 wt.%. The polymerization recipe used for the synthesis of the polyacrylic latex was the same as in Table 2, only without using the PU dispersion as the seed.

#### 2.2.3. Preparation of PU–AC Hybrids via Seeded Emulsion Polymerization

**Scheme 2:**
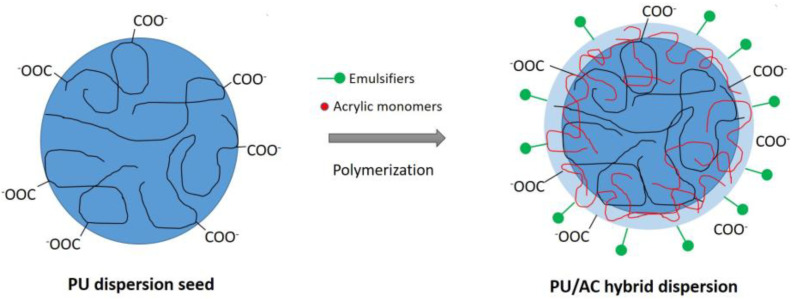
Illustration of the PU–AC hybrid synthesis via seeded emulsion polymerization.

For the synthesis of hybrid latex, a predetermined amount of PU dispersion was placed in the reactor. The ratio of PU to total acrylic monomers varied, with ratios of 1:4, 1:3, and 1:2 (S1–3), respectively, based on the mass ratio. The mixture of emulsifiers, NaHCO_3_, and a portion of water were added in the reactor and mixed. The temperature was raised to 80 °C, and the monomer mixture (MMA, BA, and MAA) and APS water solution were added dropwise in separate funnels to the reactor for 2 h. Polymerization was maintained for 90 min after completion of dripping. The reactor was cooled to room temperature and the pH was adjusted to 7.0 using triethanol amine. Finally, a stable PU–AC hybrid latex was obtained with a solid content of 25 ± 1 wt.%. The synthesis recipe is given in Table 2 and an illustration of the synthesis in Scheme 2.

#### 2.2.4. Preparation of PU–AC Hybrids via Miniemulsion Polymerization

**Scheme 3:**
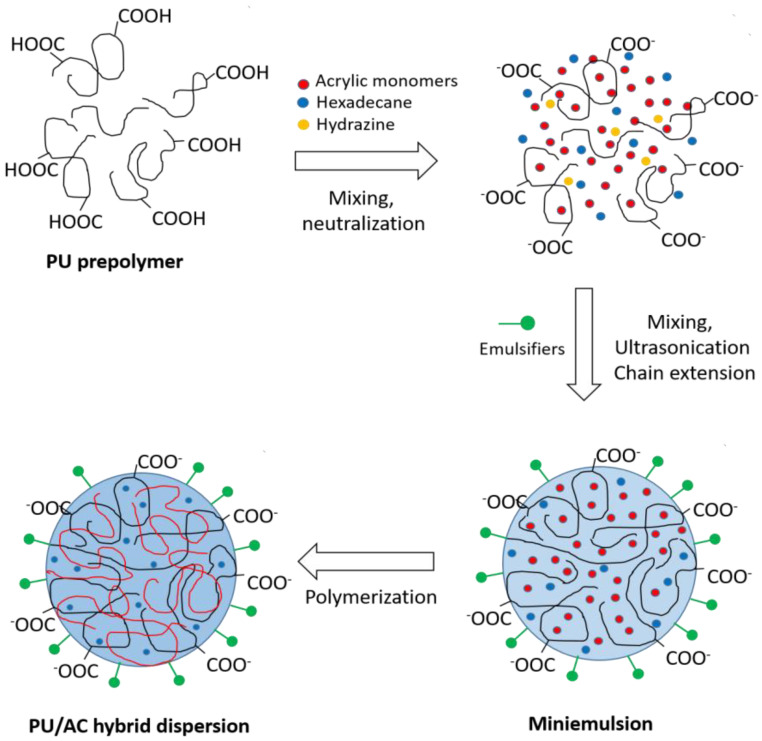
Illustration of the PU–AC hybrid synthesis via miniemulsion polymerization.

For miniemulsion polymerization, PU prepolymer, acrylic monomers (BA, MMA, and MAA), and hexadecane (HD) were mixed in an Erlenmayer. The weight ratio of PU prepolymer to total acrylic monomer was varied, with ratios of 1:3 (M1) and 1:2 (M2), respectively. The emulsifier mixture and NaHCO_3_ were dissolved in water and mixed together to obtain a pre-emulsion. Details of the ingredients are given in Table 3. The monomer/PU prepolymer mixture was then dropped into the pre-emulsion and mixed at high speed for 20 min to obtain an oil/water emulsion. The resulting emulsion was then sonicated at 70 W and 80% amplitude in an ice bath for 20 min to obtain the miniemulsion. The final emulsion was added to the reactor and heated to 80 °C. The polymerization was initiated by the addition of APS solution and reacted for 3 h. Finally, the reaction was cooled and the pH was neutralized with triethanol amine. A stable PU–AC hybrid latex was obtained. The synthesis route is given in Scheme 3. The details about all synthesized polymers were given at Table 4. 

### 2.3. Leather-Finishing Application

The synthesized PU–AC hybrid polymer emulsions were used as coating binders in leather-finishing applications to examine how their composition influences coating performance. The finishing formulation is given in Table 5. The formulation was kept simple to evaluate and compare the performance of the binders. The shoe upper crust leathers were cut to A4 sizes. The mixtures were applied by spray coating, followed by drying and hot plating at designated intervals.

### 2.4. Characterization Methods

The zeta potential values and particle size distribution of the polymer dispersions were determined using a NanoZS zetasizer (Malvern Instruments, Worcestershire, UK). Samples were prepared by diluting the latex 1:500 (v:v) with distilled water.

The chemical structure of the polymer films was identified by FTIR spectroscopy by using a Bruker ATR-FTIR spectrometer (Bruker, Billerica, MA, USA), scanning in the range of 450–4000 cm^−1^.

The thermal properties of the polymers were evaluated by Differential Scanning Calorimetry (DSC) using a TA Instruments DSC Q2000 at a heating rate of 10 °C/min under a nitrogen atmosphere, within a temperature range of −60 to 200 °C.

Performance testing of the hybrid emulsion for leather finishing involved standard methods such as flexing endurance [23], color fastness to rubbing [24], water spot resistance [25], and grain distension and strength [26]. Color changes were assessed according to grayscale standards [27,28], with ratings ranging from 1 (failure) to 5 (no color change).

The finished leathers were subjected to an accelerated aging in-house test method by exposure to UV radiation for 48 h at 60 °C in a chamber equipped with 6 UV lamps (Philips TL 20W/01 UV-B, Amsterdam, The Netherlands) and a controlled heating program. The color measurements of the leather surfaces before and after aging were read on a Konica Minolta Spectrophotometer device (model CM 3600d), and L (lightness), a (red-green color coordinate), and b (yellow-blue color coordinate) ΔE (color difference) values were determined as an average of 10 measurements.

The test for adhesion of finish was also performed on the leathers to evaluate the strength and durability of the bond between the applied coating and the leather substrate according to the official standard [29].

## 3. Results and Discussions

### 3.1. Particle Size of the Latexes

**Figure 1:**
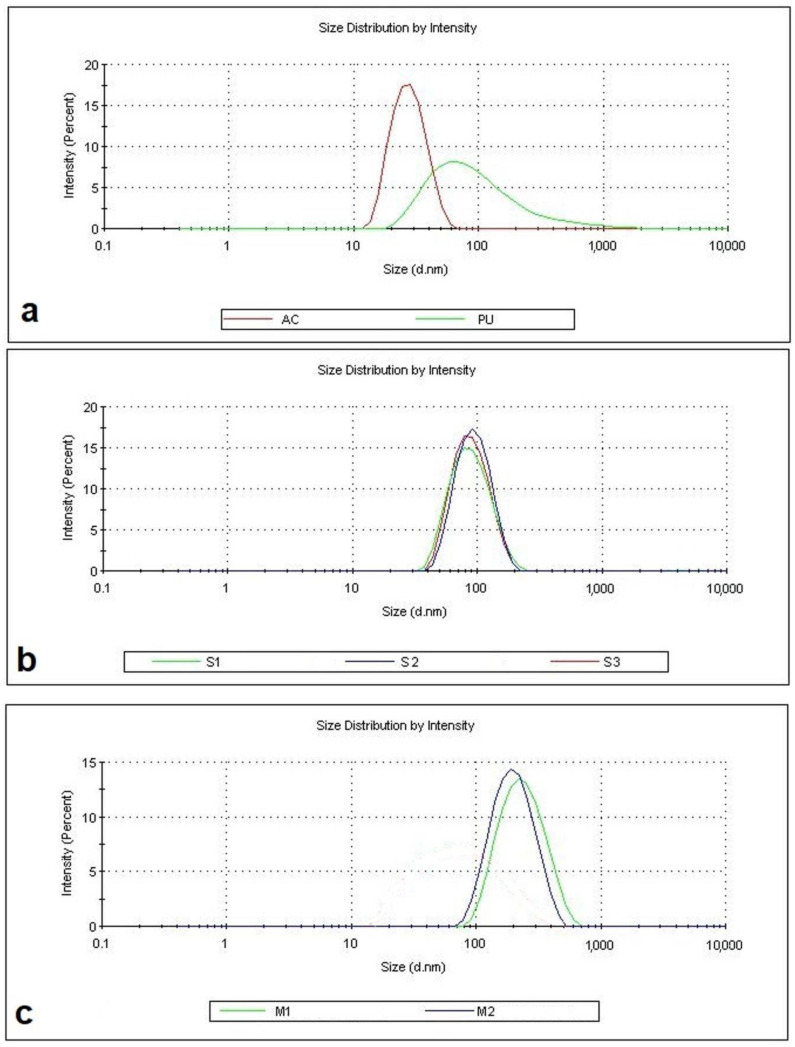
Particle size distribution curves of the latexes: (**a**) neat PU and AC latexes, (**b**) seeded hybrid latexes (S), and (**c**) hybrid miniemulsions (M).

The average particle size diameter, polydispersity index (PDI), and zeta potential values of the latexes are summarized in Table 6, and the particle size distributions are shown in Figure 1. The PU dispersion exhibited a low average particle size of 66 nm and a relatively high PDI, as expected, due to the absence of an emulsifier during the dispersion of the prepolymer in water. In contrast, the pure acrylic latex was synthesized with a very low average particle size and PDI, reflecting the effect of emulsifier use. The average particle sizes of the PU–AC hybrid latexes obtained by seeded emulsion polymerization ranged from 82 to 89 nm, which were larger than for the PU dispersion, as expected, and these latexes exhibited low PDI values. The hybrid latexes obtained by miniemulsion polymerization (M1 and M2) had significantly larger particle sizes of 215 nm and 178 nm, respectively.

Because the original miniemulsion droplets are formed by a shearing process (e.g., ultrasound), the droplet size distribution is typically broad, leading to large polydispersity in the resulting particle size distributions [19,30]. The application of high shear (e.g., ultrasound) has a significant effect on particle size and distribution. A potential issue with ultrasound-based systems is that only a small region around the sonifier tip is directly affected by ultrasound waves, while other regions may experience less shear force. This may contribute to larger particle sizes and broader distributions [31]. In addition to the homogenization effectiveness, the use of emulsifiers, water soluble initiation systems, and hydrophilic monomers also affects particle size and final polymer properties in miniemulsion systems. When the emulsifier concentration exceeds the critical micelle concentration (CMC) and hydrophilic monomers are used, secondary particle nucleation can occur from micelles near the droplet nucleation sites [32,33,34,35].

The use of water-soluble initiators instead of oil-soluble ones further contributes to the formation of secondary particle nucleation systems. Peixoto et al. (2016) investigated secondary particle nucleation in miniemulsion polymerizations of methyl methacrylate in the presence of hydrophilic monomers (e.g., acrylic acid, methacrylic acid, 2-hydroxyethyl methacrylate, and methacrylamide) and water/oil initiators (KPS/BPO) [36]. They reported that when a water-soluble initiator was used with hydrophilic monomers, secondary particle nucleation was inevitable, leading to variations in average particle sizes, size distributions, and molecular weights. Considering the use of the hydrophilic monomer MAA, water-soluble initiator (APS), and emulsifiers in our study, secondary particle nucleation likely occurred, resulting in larger particle sizes and relatively higher PDI values.

Despite the larger particle sizes, these latexes remained stable with low PDI values (0.125 and 0.133). Figure 1 illustrates the size distributions of the latexes, all of which display a monomodal distribution with a single peak and relatively low PDI, confirming the uniformity of the hybrid particle structure. This indicates that the acrylic and PU segments formed a single, homogeneous dispersion without phase separation. The zeta potential values of the latexes were also presented in Table 6. The zeta potential indicates the degree of repulsion between adjacent, similarly charged particles in a dispersion. For molecules and particles that are small enough, a high zeta potential will confer stability, i.e., the solution or dispersion will resist aggregation. Dispersions with zeta potential values between ±40 and ±60 mV indicate good stability, while those above ±61 mV exhibit excellent stability [37]. The zeta potentials of the PU and AC dispersions were found to be −53.7 mV and −51.2 mV, respectively, indicating good stability. The hybrids obtained from seeded emulsion polymerization exhibited excellent stability, with zeta potential values ranging from −68 to −79 mV. This was likely due to the higher number of negatively charged functional groups contributed by both emulsifiers and PU chains in the seeded emulsion process.

On the other hand, the zeta potential values of the miniemulsions (M1: −53.5 mV, M2: −46.5 mV) were higher than those of the seeded emulsions, possibly due to their larger particle sizes and/or lower number of particles (solid content 20%). Nevertheless, their zeta potential values were still under −46 mV, indicating good stability.

### 3.2. FTIR Analysis

**Figure 2:**
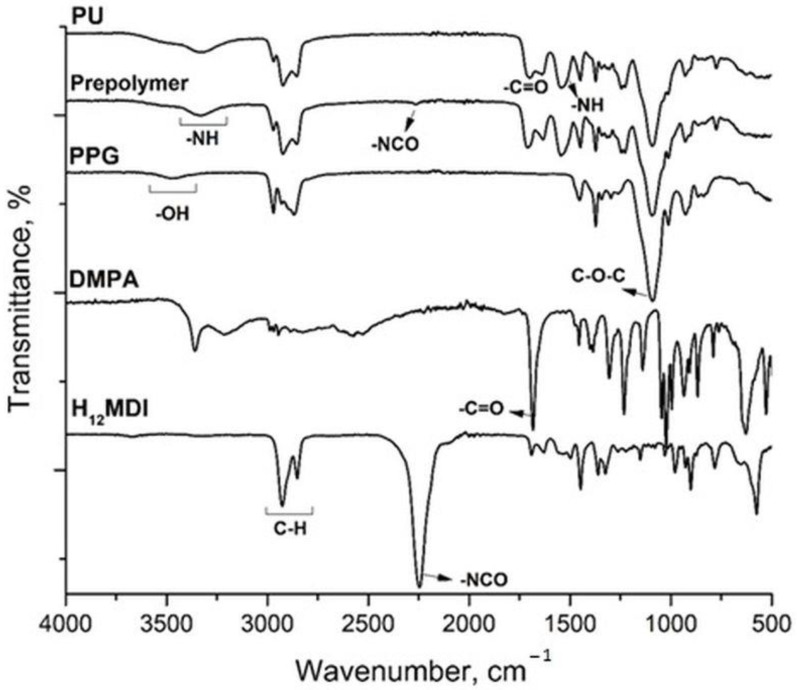
FTIR spectra of the PU, prepolymer, and PU monomers.

The structure of the polymers was evaluated by performing FTIR analysis and by using FTIR databases to interpret the spectra. The characteristic absorption bands of the starting materials, the prepolymer, and the final PU are shown in Figure 2. The monitoring of PU formation was assessed by following the intensity of the isocyanate(-NCO) band. The -NCO band of the diisocyanate decreased to a small band due to the urethane reaction in the prepolymer and completely disappeared in the final PU spectrum. Other characteristic bands of PU were observed at 3500 and 3326 cm^−1^, which can be attributed to –OH and N–H stretching; at 2970, 2922, and 2855 cm^−1^, corresponding to C–H stretching; at 1700 and 1638 cm^−1^, indicating C=O stretching; at 1545 cm^−1^, corresponding to N–H bending; at 1245 and 1248 cm^−1^, related to C–N stretching; and at 1090 cm^−1^, corresponding to C–O–C stretching. IR analysis confirmed the expected structure of the synthesized polyurethane.

**Figure 3:**
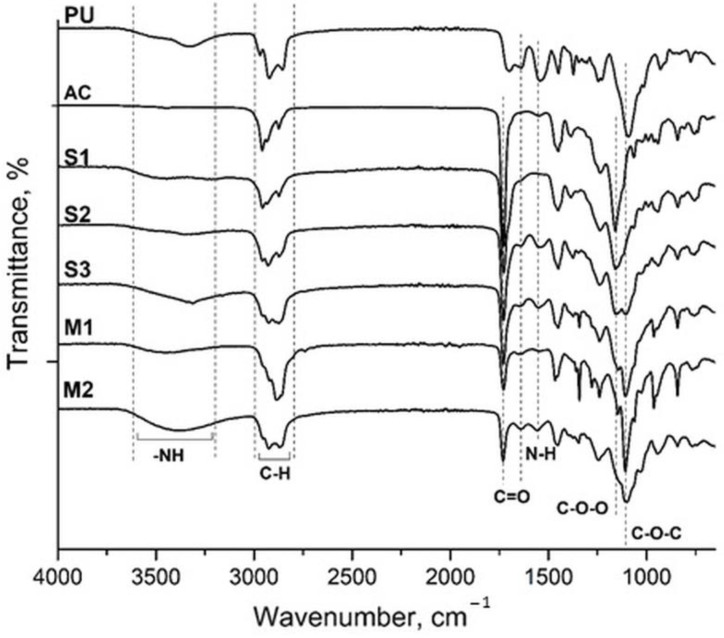
FTIR spectra of the PU, AC, and PU–AC hybrid polymers.

Figure 3 presents the FTIR spectra of the hybrid polymers alongside pure polyacrylic and PU polymers. The characteristic bands of the acrylic polymer, composed of BA, MMA, and MAA as monomers, were observed at 3447 cm^−1^, corresponding to –OH stretching (COOH); at 2958, 2934, and 2874 cm^−1^, attributed to C–H stretching; at 1730 cm^−1^, for C=O stretching; at 1449 cm^−1^, for –CH bending; and at 1160 cm^−1^, for C–O (ester) stretching vibrations. The FTIR spectra of the hybrid polymers showed characteristic absorption bands from both PU and AC polymers. N–H stretching was observed between 3600 and 3100 cm^−1^, C–H stretching at 2850–2970 cm^−1^, C=O stretching of the acrylic group at 1733 cm^−1^, C=O stretching of PU around 1637 cm^−1^, –NH bending at 1552 cm^−1^, C–O–O stretching at 1156 cm^−1^, and C–O–C at 1100 cm^−1^. As the weight ratio of PU to AC increased, the intensity of the corresponding absorption bands also increased proportionally. These bands are marked in Figure 3. Overall, the FTIR analysis confirmed the successful synthesis of the hybrid polymers.

### 3.3. DSC Analysis

**Figure 4:**
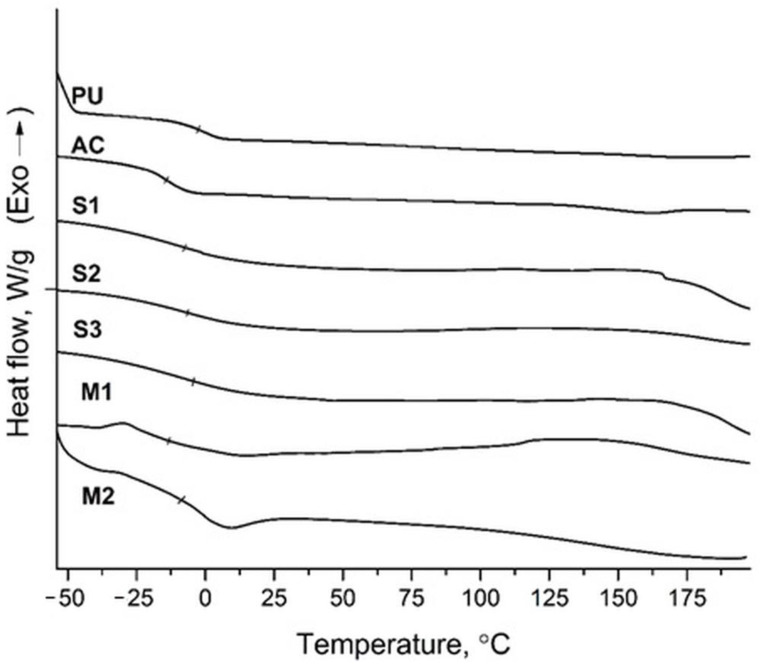
DSC thermograms of PU, AC, and hybrid polymers.

DSC analysis was conducted to investigate the thermal transitions of the polymer films; the thermograms are shown in Figure 4.The glass transition temperatures (T_g_) of the pure and hybrid polymers were determined from these thermograms. The glass transitions of pure PU and AC were clear and the T_g_ values were found to be −2.5 °C and −14.2 °C, respectively. For the hybrid polymers obtained by seeded emulsion polymerization (S1–3), the glass transition was broader, probably due to the homogenous distribution of PU and AC polymer chains within each other. The calculated T_g_ values for these hybrids were found to be −7.6 °C, −6.4 °C, and −4.4 °C for S1, S2, and S3, respectively, being between those of the pure AC and PU polymers. The glass transitions of the hybrid polymers obtained via miniemulsion polymerization (M1 and M2) were also slightly wider, although the transition was more pronounced. The T_g_ values of these hybrids were −13.3 °C and −8.8 °C, respectively, again laying between the T_g_s of pure AC and PU polymers. In the thermograms, a single glass transition was observed for all the hybrid polymers. This may be due to the close T_g_ values of pure PU and AC, as well as the good compatibility and enhanced interaction between the polymer chains of PU and AC, supporting the presence of a single T_g_. Additionally, for all hybrid polymers, a slight increase in T_g_ values was observed as the PU ratio increased.

### 3.4. Leather-Finishing Performance

**Figure 5:**
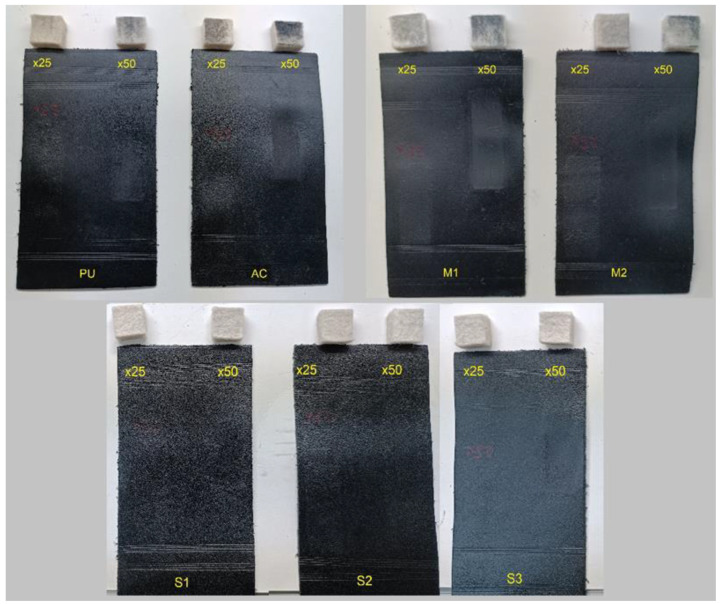
Images of the finished leather samples after the wet rubbing test.

The results of physical tests performed on the finished leathers to evaluate the coating performance of the polymers are presented in Table 7, Table 8 and Table 9. One of the key criteria used to assess the coating performance is the transfer of color from the dyed leather surface to an adjacent area (e.g., cotton, felt, etc.) primarily through rubbing. In this test, a white felt (either dry or wet) is loaded with weight and rubbed repeatedly over a stretched leather surface. Both the leather and the felt are then examined for any changes. The felt is tested to determine if any paint has rubbed off of the leather. The rubbing test results are provided in Table 7. The rubbing test was initially run 100 times in dry conditions, with results ranging from an average score of 3 to a good score of 5 for most leathers. Therefore, the test was extended to 500 rubs. As can be seen from the results, the leather coated with pure polyacrylic showed values of 3 and 4 for leather and felt, respectively, while the PU-coated leather showed slightly higher values of 3/4 and 4 for leather and felt. Leathers coated with hybrid polymers obtained by the seeded emulsion technique showed higher grayscale values than the control samples, with no color transfer to the felt. Furthermore, the difference in performance was more pronounced during wet rubbing (Figure 5). Polyacrylic-coated leather showed complete failure (1), and the PU-coated leather showed very low values (1/2 to 2) after 50 cycles of wet rubbing. However, hybrid polymers (S1–3) showed significantly higher values, ranging from 3/4 to 4/5. It was also observed that increasing the PU ratio resulted in better rubbing performance. On the other hand, the performance of leathers coated with hybrid polymers obtained by miniemulsion polymerization was slightly lower than that of the control leathers, especially for M1. The M2-coated leather sample showed slightly higher values than the polyacrylic-coated leather, but lower than pure PU for the rubbing test.

Another important test, particularly for shoe upper leathers, is flexing endurance, which involves subjecting leather samples to repeated cycles of flexing and bending to assess their resistance to cracking and other forms of damage. The results are summarized in Table 8. Initially, the test was carried out for 20,000 flexing cycles, with no visible damage observed for any of the samples. The test was further extended to 50,000 cycles. The evaluation showed no failure or color change for any of the leathers. However, some wrinkles were observed due to the flexing action, being more pronounced on the PU and hybrid-coated leathers.

The water spotting test assesses the resistance of the leather surface to water-related effects such as marking, staining, blistering, and color change. This test helps evaluate how shoe care treatments (such as polishing or protective sprays) influence the material’s ability to resist stains. It can also be used to test the inner surface of patent leather and other plastic-coated leathers by wetting them. Two drops of distilled water were applied to the leather surface during the test. Excess water from one drop was removed using filter paper, and any physical effects were noted after 30 min. The second drop was allowed to evaporate overnight, and any color changes were evaluated using standard grayscale. The results are summarized in Table 9. The findings showed that water was partially absorbed by AC, PU, M1, and M2, whereas S1–3 completely absorbed the water droplet after 30 min. No physical damage or noticeable changes were observed after the first drop was removed. No significant color changes were observed after 16 h, except for light spots on M2- and PU-coated leathers. Overall, most leathers performed well in the water spotting test.

Table 10 presents the strength and distention values of the leathers obtained from the ball burst test. This test measures the mechanical properties of leather by evaluating its resistance to deformation and rupture under stress. A ball-shaped probe applies force until the leather bursts, providing key data such as bursting strength, elongation at burst, and tear resistance. This test is essential for assessing the durability, flexibility, and surface integrity of leather, particularly for applications like footwear and upholstery. While bursting strength is primarily influenced by the internal fiber structure of the leather, surface integrity, strength of the finish layer, and elasticity affect its cracking and bursting behavior. The results showed that grain cracking strength was considerably higher for the hybrid samples, especially for S1–3, compared to the control leathers. The grain cracking strength of AC and PU increased from 5.0–7.5 kg to 13–20 kg for seeded hybrids and to 6.5–8.0 kg for miniemulsion hybrids. Similarly, the strength at grain burst was higher for the hybrid polymers. The distension values were comparable. Overall, the results indicate that hybrid polymers exhibited better mechanical performance than the pure polymers.

Adhesion test results for finished leathers were also presented in Table 10. Adhesion testing evaluates the strength of the bond between a coating or finish and the leather surface and provides information on the durability and performance of coatings or finishes applied to leather products. The peeling strength values of the AC- and PU-finished control leathers were found to be 5.5 and 6.3 N, being improved for all hybrid polymers. Like the rubbing and ball burst results, the increase in adhesion strength was more pronounced for leathers coated with seeded hybrid dispersions (S1–3) which showed values ranging from 8.4 N to 10.2 N. Given that the minimum adhesion strength generally accepted for leather products is 2 N, all leather samples showed excellent adhesion strength.

To evaluate the long-term stability of the leathers under environmental factors, the finished leather samples were subjected to an accelerated aging test by exposing them to increased temperature (70 °C) for 48 h under UV radiation. The color of the leather samples was measured before and after the test, and the values of the colorimetric coordinate values (L, a, b) and color differences (ΔE) of the finished leathers are presented in Table 11. Usually, exposure to heat and UV radiation would lead to the fading of color intensity (+L) or to an increase in yellow hue (+b). However, the results indicated that the color coordinates showed no significant changes for any of the leather samples. Considering that the color difference scale (ΔE) ranges from 0 to 100, the ΔE values were very small, suggesting that the finish coating exhibited high aging resistance. Pigment color is also known to influence aging behavior. Since black pigments are known for their high resistance to aging, further investigations on the aging behavior, particularly using different colored pigments, would provide more detailed information on the aging properties.

Upon evaluating the overall test results, it was observed that leathers coated with PU–AC hybrid polymer dispersions exhibited superior performance compared to those coated with pure acrylic (AC) or polyurethane (PU) dispersions. Performance differences were particularly evident in rubbing fastness, grain strength, and adhesion strength, with the seeded hybrid polymers showing the most pronounced improvements. The enhanced performance of the seeded hybrids can probably be attributed to their smaller particle size and the improved integrity of the acrylic and urethane domains, compared to the miniemulsion hybrids.

Particle size is a crucial parameter influencing the performance of coatings [1,38]. In seeded emulsion polymerization, the number of particles is generally higher than in miniemulsion systems. A larger number of particles results in a lower concentration of radicals per particle, which reduces termination events and facilitates longer polymer chain growth, ultimately increasing the degree of polymerization. Furthermore, smaller particles have a higher surface area-to-volume ratio, which promotes better coalescence and smoother film formation during the drying process. In leather-finishing applications, smaller particles can penetrate more effectively, resulting in improved adhesion, better pigment binding, and enhanced coverage.

While the miniemulsion polymerization system may seem more suitable for synthesizing hybrid polymers, it is highly sensitive to various factors, making it dependent on each specific system. Factors such as the localized effect of ultrasonication, the use of hydrophilic monomers, the concentration of emulsifier, and other variables can influence the particle size, distribution, nucleation, and, consequently, the degree of polymerization and the monomer composition of the polymer chains. In this study, the seeded emulsion polymerization system produced more uniform and smaller particle-sized hybrids, resulting in better coating performance. However, further structural and morphological investigations are needed to gain a deeper understanding of this phenomenon, which will be the subject of future research.

## 4. Conclusions

Acrylic–urethane hybrid polymer dispersions were successfully synthesized using both seeded emulsion and miniemulsion polymerization methods and subsequently characterized. Both synthesis methods resulted in stable and uniform dispersions; however, hybrids created by seeded emulsion polymerization showed smaller particle sizes and higher zeta potential values. Characterization by FTIR and DSC confirmed the strong compatibility between the polymer phases, resulting in single-phase thermal transitions and consistent chemical properties.

When used as binders in leather-finishing formulations, the hybrids have shown significant performance improvements compared to their pure acrylic and urethane counterparts. Leather samples coated with hybrid emulsions demonstrated superior flexing endurance, withstanding up to 50,000 cycles without significant cracking or color changes. In addition, the hybrids exhibited increased rubbing fastness, improved grain cracking resistance, and enhanced adhesion strength.

Among the two synthesis methods, seeded emulsion polymerization provided superior coating performance compared to miniemulsion polymerization. This is likely due to the more stable, homogeneous dispersions with smaller particle sizes produced by the seeded emulsion process, which also enhanced phase compatibility and integrity. The wet rubbing performance of the seeded hybrids was almost as high as that of crosslinked coatings obtained using external crosslinking agents. This performance is likely due to the strong chain entanglements formed by the unevenly distributed PU and AC domains, making the hybrid a complex, physically crosslinked polymer. While miniemulsion polymerization offers advantages such as the ability to encapsulate reactive components, its performance is influenced by various factors (e.g., efficiency and homogeneity of emulsification, secondary nucleation) that may limit its effectiveness in coating applications.

From an industrial perspective, the seeded emulsion polymerization method is more feasible and economically viable for large-scale production. This method is widely used in the production of latex binders and water-based adhesives due to its precise control of particle size and heat management during exothermic radical reactions. In contrast, miniemulsion polymerization requires high-energy emulsification processes, such as sonication, which complicates large-scale industrial applications. Furthermore, miniemulsion polymerization typically relies on batch systems, making heat management during the reaction more difficult. Thus, the synthesis of PU–AC hybrid systems using seeded polymerization is more suitable for industrial-scale production.

Overall, this study highlights the benefits of acrylic–urethane hybrid dispersions as versatile and high-performance binders. Acrylic binders are significantly more cost-effective than polyurethane dispersions, primarily due to the lower cost and wide availability of monomers (acrylic binders: 1.8–2.5 USD/kg, polyurethane dispersions: 5–7.5 USD/kg). This cost advantage makes hybrid polymers an attractive alternative. For instance, a hybrid polymer with an acrylic-to-polyurethane ratio of 2:1 (e.g., S3) not only demonstrates superior performance but also achieves a remarkable cost reduction of nearly 50% compared to conventional polyurethane dispersions. Considering these advantages, it is evident that urethane–acrylic hybrids represent a highly promising class of materials with significant potential for leather-finishing applications.

## Figures and Tables

**Table 1:** The recipe used for the PU synthesis.

Components	Amount (g)
PPGs	12.74
H_12_MDI	9.80
DMPA	0.98
Catalyst	0.01
DME	6.72
Hydrazine	0.84
Water	68.90
Total	100.00

**Table 2:** The recipe used for the synthesis of urethane–acrylic hybrid dispersions via seeded emulsion polymerization.

Components	Amount (g)
PU dispersion (seed)	31.31
BA	9.57
MMA	5.22
MAA	0.87
Emulsogen EPN 287	1.30
Sulfopon^®^ 101 UP	1.63
NaHCO_3_	0.02
APS	0.09
Water	50.00
Total	100.00

**Table 3:** The recipe used for the synthesis of urethane–acrylic hybrid dispersions via miniemulsion polymerization.

Components	Amount (g)
PU prepolymer	6.02
BA	7.36
MMA	4.01
MAA	0.67
HD	0.94
Hydrazine	0.15
Emulsogen EPN 287	1.00
Sulfopon^®^ 101 UP	1.25
NaHCO_3_	0.01
APS	0.07
Water	78.52
Total	100.00

**Table 4:** Details of the synthesized polymers.

Sample	Technique	AC Content (Parts by Weight)	PU Content (Parts by Weight)	Total Solid Content (wt.%)	Final pH	Conversion (%)
PU	Urethane reaction + chain extension in water	-	1	25 ± 1	8.0 ± 0.5	>99.0
AC	Seeded emulsion polymerization	1	-	25 ± 1	7.0 ± 0.5	>99.0
S1	Seeded emulsion polymerization	4	1	25 ± 1	7.0 ± 0.5	>98.5
S2	Seeded emulsion polymerization	3	1	25 ± 1	7.0 ± 0.5	>98.0
S3	Seeded emulsion polymerization	2	1	25 ± 1	7.0 ± 0.5	>98.0
M1	Miniemulsion polymerization	3	1	20 ± 1	7.0 ± 0.5	>97.0
M2	Miniemulsion polymerization	2	1	20 ± 1	7.0 ± 0.5	>97.5

**Table 5:** The finish formulation applied on the leathers.

Components	Application Steps		Descriptions
	Basecoat (I) (Parts)	Topcoat (II) (Parts)	
Water	55	35	Spray I × 3 times
Pigment	10	-	Hot plate 90 °C/100 bar
Wax	5	-	
Polymer emulsion	30	-	Spray I × 2 times
Isopropyl alcohol	0.5	-	Hot plate 90 °C/70 bar
Aqueous NC lacquer	-	15	Spray II × 1 time

**Table 6:** The average particle sizes and size distributions of the latexes.

Polymer Sample	Average Particle Diameter (nm)	Polydispersity Index (PDI)	Zeta Potential Values (mV)
PU	66.11 ± 5.59	0.275 ± 0.070	−53.70 ± 5.43
AC	29.77 ± 6.06	0.075 ± 0.014	−51.20 ± 9.73
S1	82.77 ± 6.22	0.134 ± 0.013	−69.80 ± 5.49
S2	89.32 ± 5.77	0.068 ± 0.019	−68.10 ± 7.79
S3	86.63 ± 1.58	0.118 ± 0.014	−79.30 ± 6.16
M1	215.00 ± 1.19	0.133 ± 0.006	−53.50 ± 9.14
M2	178.13 ± 2.12	0.125 ± 0.007	−46.50 ± 13.70

**Table 7:** Rubbing fastness properties of leather samples coated with hybrid latexes.

Samples	Evaluated Parts	Gray Scale Values			
		Dry		Wet	
		100 Cycles	500 Cycles	25 Cycles	50 Cycles
AC	Leather	3/4	3	3	1
	Felt	4/5	4	1	1
PU	Leather	4	3/4	4	1/2
	Felt	5	4	3	2
S1	Leather	4/5	4	3/4	3/4
	Felt	5	5	4/5	4
S2	Leather	4/5	4	4	3/4
	Felt	5	5	4/5	4/5
S3	Leather	4/5	4	4	4
	Felt	5	5	5	4/5
M1	Leather	2/3	1/2	3	1
	Felt	3/4	2	1/2	1
M2	Leather	3/4	3	3	1/2
	Felt	4/5	4	2	1/2

**Table 8:** Observations following the flexing endurance tests of finished leathers.

Samples	Flexometer	
	X 20.000	X 50.000
AC	Very light wrinkles	Light wrinkles
PU	Light wrinkles	Deep wrinkles
S1	Light wrinkles	Deep wrinkles
S2	Light wrinkles	Deep wrinkles
S3	Light wrinkles	Deep wrinkles
M1	Light wrinkles	Deep wrinkles
M2	Light wrinkles	Deep wrinkles

**Table 9:** Color fastness of leather samples to water spotting after 30 min and 16 h.

	30 min		16 h	
Leathers	Observation	Gray Scale Value	Observation	Gray Scale Value
AC	The water drop was partially absorbed; no spot after drying.	5	No spot	5
PU	The water drop was partially absorbed; very light spot.	4/5	Very light spot	4/5
S1	Completely absorbed by leather; no spot.	5	No spot	4/5
S2	Completely absorbed by leather; no spot.	5	No spot	5
S3	Completely absorbed by leather; no spot.	5	No spot	5
M1	The water drop was partially absorbed; no spot.	5	No spot	5
M2	The water drop was partially absorbed; very light spot after drying.	4/5	Light spot	4

**Table 10:** Results obtained from the ball burst test (distension and strength of grain).

Samples	Grain Cracking		Grain Burst		Adhesion of Finish
	Strength (kg)	Distension (mm)	Strength (kg)	Distension (mm)	Strength (N)
AC	5.0 ± 1.2	71.0 ± 0.2	20.5 ± 0.5	99.8 ± 1.7	5.5 ± 0.92
PU	7.5 ± 0.5	68.9 ± 0.4	24.5 ± 0.5	98.0 ± 2.0	6.3 ± 1.80
S1	13.0 ± 2.0	64.3 ± 2.3	26.0 ± 1.0	81.3 ± 1.3	9.8 ± 1.42
S2	18.0 ± 0.5	75.5 ± 1.0	26.0 ± 2.0	87.6 ± 0.2	8.4 ± 2.10
S3	20.0 ± 2.0	72.5 ± 0.5	30.0 ± 2.0	83.2 ± 0.2	10.2 ± 3.24
M1	8.0 ± 0.5	69.9 ± 0.2	25.5 ± 2.5	97.8 ± 1.9	6.4 ± 0.77
M2	9.0 ± 2.0	64.9 ± 0.2	33.0 ± 4.0	99.0 ± 0.8	7.1 ± 1.25

**Table 11:** The colorimetric coordinate values of the leathers before (1) and after (2) 48 h aging test.

Samples	L1	L2	a1	a2	b1	b2	ΔE
AC	24.630	24.628	−0.018	−0.022	−0.606	−0.674	0.068
PU	24.358	24.908	0.024	−0.002	−0.636	−0.618	0.551
S1	24.306	24.900	−0.030	−0.066	−0.700	−0.762	0.598
S2	24.292	24.578	−0.002	−0.024	−0.636	−0.696	0.293
S3	24.350	24.678	−0.024	−0.036	−0.712	−0.768	0.333
M1	24.612	24.432	0.014	−0.016	−0.558	−0.724	0.247
M2	24.232	24.494	−0.026	0.022	−0.572	−0.684	0.289

## Data Availability

The data that support the findings of this study are available on request from the corresponding author.
